# A fast open-source Fiji-macro to quantify virus infection and transfection on single-cell level by fluorescence microscopy

**DOI:** 10.1016/j.mex.2022.101834

**Published:** 2022-09-02

**Authors:** Yannic Kerkhoff, Stefanie Wedepohl, Chuanxiong Nie, Vahid Ahmadi, Rainer Haag, Stephan Block

**Affiliations:** Institute of Chemistry and Biochemistry, Freie Universität Berlin, Berlin, Germany

**Keywords:** Automatic cell segmentation, Single-cell analysis, Quantitative fluorescence microscopy, Infection inhibition, Transfection efficiency

## Abstract

The ability to automatically analyze large quantities of image data is a valuable tool for many biochemical assays, as it rapidly provides reliable data. Here, we describe a fast and robust Fiji macro for the analysis of cellular fluorescence microscopy images with single-cell resolution. The macro presented here was validated by successful reconstruction of fluorescent and non-fluorescent cell mixing ratios (for fluorescence fractions ranging between 0 and 100%) and applied to quantify the efficiency of transfection and virus infection inhibition. It performed well compared with manually obtained image quantification data. Its use is not limited to the cases shown here but is applicable for most monolayered cellular assays with nuclei staining. We provide a detailed description of how the macro works and how it is applied to image data. It can be downloaded free of charge and may be used by and modified according to the needs of the user.

• Rapid, simple, and reproducible segmentation of eukaryotic cells in confluent cellular assays

• Open-source software for use without technical or computational expertise

• Single-cell analysis allows identification and quantification of virus infected cell populations and infection inhibition

## Graphical abstract

**Figure:**
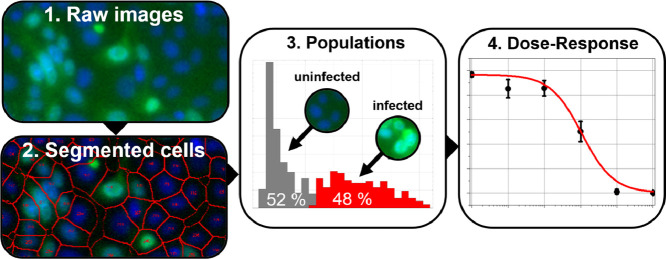

## Specifications table

**Table:** 

Subject area;	
More specific subject area;	Automatic single-cell segmentation
Name of your method;	Single-cell fluorescence quantification (SCFQ) macro
Name and reference of original method;	*NA*
Resource availability;	Fiji is freely available (https://imagej.net/software/fiji/downloads).
	The macro (S7), a tutorial video (S8) and widefield images from the cell mixing experiment (S9) are provided as free separate supplementary materials

## Introduction

Cellular infection assays are a widely used laboratory method to probe the infectivity of viruses as well as to test potential virus infection inhibitors [1], and proved to be highly valuable especially in times like the current SARS-CoV-2 pandemic [2]. Many infection assays can be analyzed by imaging an infected cell culture using optical microscopy, by staining of the cell nuclei (*e.g.*, using Hoechst 33342) and by using a fluorescent marker (*e.g.*, GFP expression [3]) or immunostaining of viral proteins [4]. To show a general effect of infection or infection inhibition, the acquired images are often examined only qualitatively. However, an image series of different experimental conditions (*e.g.*, treatment with different inhibitor concentrations) can also provide quantitative information [5] (*e.g.*, the inhibitor potency based on the IC_50_ value) if one is able to extract this information from the image series. In recent years, several open-source software solutions have been developed that allow to extract image-based fluorescence information with single-cell resolution [6], [7], [8], [9], [10], [11], [12]. Each of these software solutions have different analysis strategies and aim for different fluorescence readouts. For example, QuantIF [9] and FNMM [11] aim for the colocalization of the nucleus staining with another fluorescence signal. FluoQ [6] and PiQSARS [12] are tailored for time-lapse-based experiments, while Cytokit [8] is specialized in correlating single-cell parameters with spatial information (for details see Supplemental Materials Section S6: Image analysis software overview). However, as most software is highly specialized for specific tasks, the software solutions mentioned above may not necessarily meet the needs of all users. This is due to the high variability of experimental setups (*e.g.*, the microscopy method, magnification, cell type, and cell density used) and read-outs (*e.g.*, fluorescence source, intensity, and fraction) used by the community. To address this, we developed and validated a versatile, easy-to-use and open-source Fiji macro [13] that is capable of quantifying transfection, viral infection, or inhibition of viral infection by evaluating intensity distributions at the level of individual cells in monolayered cellular assays. The macro was optimized to perform on a wide range of cell densities and fluorescent cell fractions for different cell lines as well as experimental and microscopy setups. It requires only three input parameters (addressing background signal, cell density, and marker intensity) and allows for fast batch analysis with detailed single-cell information. Besides fluorescence, the position, size, and circularity of each detected cell is saved in a data table, which allows, *e.g.*, for the localization of fluorescent and non-fluorescent cells within the sample. It contains an automatic correction of background fluorescence and provides a segmentation overlay and visualization of cell populations for easy manual inspection of the analysis output.

**Fig. 1:**
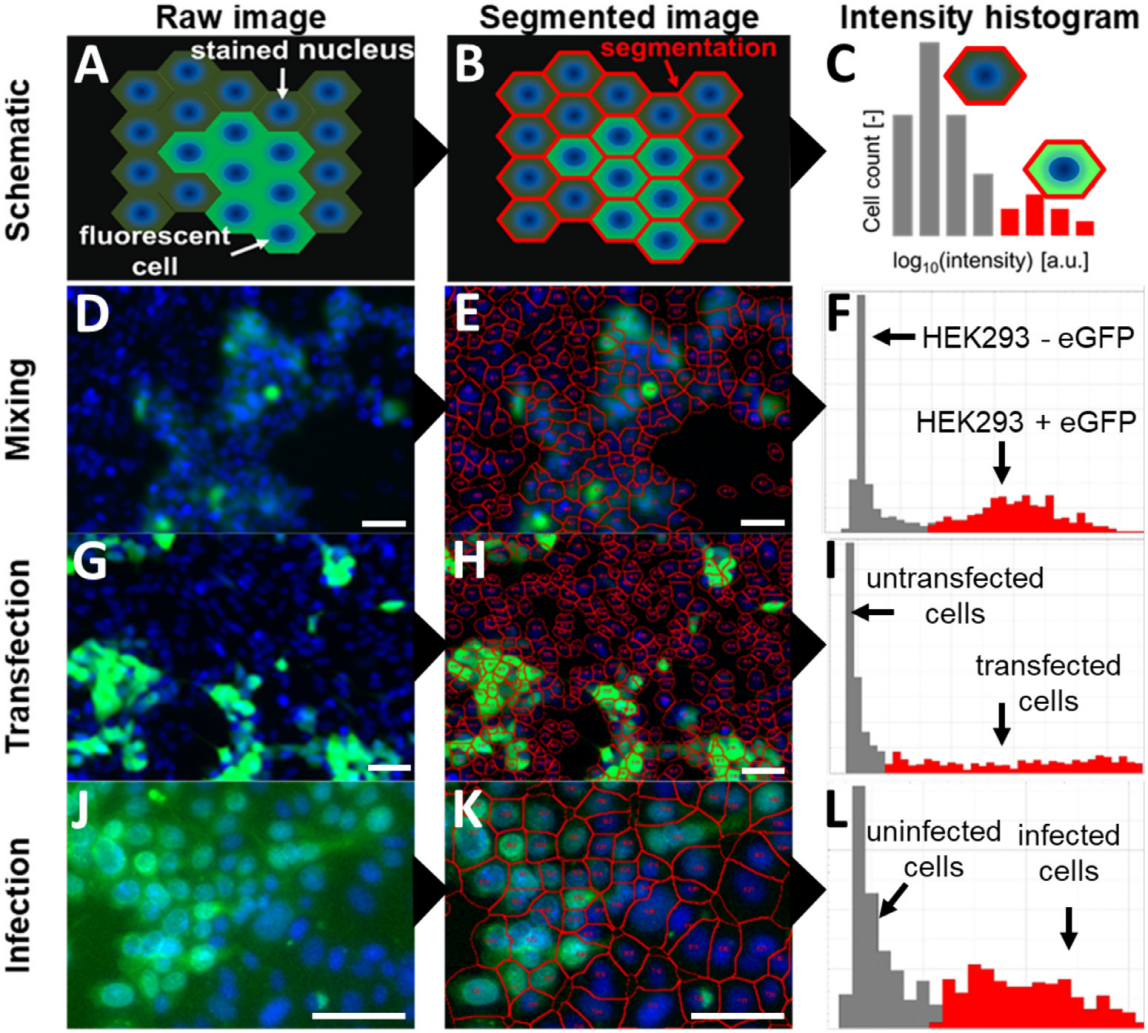
Concept overview (scale bars = 50 µm) of the single-cell fluorescence quantification procedure. The raw images (A, D, G, J) consist of two-dimensional cell monolayers, in which the nuclei of all cells have been stained (blue), while only a fraction of the cells shows a fluorescent signal of a fluorescent marker (green). Individual cells are identified based on their nucleus (centers), segmented by a watershed algorithm (red lines, B, E, H, K), and separated from empty (cell-free) areas by their intrinsic autofluorescence signal. Calculating log_10_ intensity histograms (C, F, I, L) of the observed single-cell fluorescence values typically reveals two populations, which correspond to fluorescent (red) and non-fluorescent cells (gray) observed in the image.

Fig. 1 shows an overview of our approach for quantifying fluorescent cell populations. First, images of a cell monolayer with stained nuclei and another fluorescent marker (dependent on the experimental setup) are acquired (Fig. 1A, D, G, J). The cells are automatically segmented (Fig. 1B, E, H, K) and the fluorescent marker intensity is quantified for each cell individually, enabling to discriminate between different populations (recognizable as two peaks or one peak with a tail in Fig. 1C, F, I, L) across the cell ensemble.

## Method details

### Cell transfection and mixing

10^4^ human embryonic kidney cells (HEK293, #ACC 305, Leibnitz Institute DSMZ – German Collection of Microorganisms and Cell Cultures GmbH) per well were seeded in full medium (DMEM supplemented with 10% FBS (#P04-04500 and #P30-3031, PAN Biotech Germany), 0.1 g/L streptomycin sulfate and 0.065 g/L penicillin G potassium (#1852,0100 and #A1837,0100, BioChemica Germany)) into 96 well plates and incubated over night at 37 °C and 5% CO_2_. On the next day, transfection complexes were formed and added to the cells as follows: 0.1 µg plasmid DNA (pEGFP-N3, Clontech) was diluted in 10 µL 150 mM NaCl solution (saline) and 0.1 to 0.6 µg of PEI (25 kDa branched, #408727, Sigma-Aldrich) was diluted in 10 µL of saline separately. The PEI dilutions were added to the DNA dilutions under vigorous mixing for 5–10 s and incubated for 15 min at room temperature afterwards. The cell culture supernatant was replaced with 100 µL/well fresh medium and 20 µL/well of transfection complexes were added. After incubation for 48 h at 37 °C and 5% CO_2_, cell nuclei were stained with Hoechst 33342 (1 µg/mL in medium, #H1399, Thermo Fisher Scientific) for 10 min at 37 °C. The cell culture supernatant was replaced with fresh medium and cells in the plates were imaged with a 10x objective (A-plan 10x/0.25 Ph1, #441031-9910, Zeiss; FOV: 895.26 µm x 670.80 µm) on a Zeiss Axio Observer Z1 widefield fluorescence microscope equipped with an Illuminator HXP 120C, Colibri LED light sources 400, 530 and 625 nm, and an AxioCam MRm monochrome CCD camera. The ZEN software was used for image acquisition with the default GFP, DAPI or phase contrast settings using filter sets 38 (GFP: excitation 450–490 nm, emission 500–550 nm) and 49 (DAPI: excitation 335–383 nm, emission 420–470 nm). The confocal images of Fig. 3B were taken with a 20x objective (20x/0.75 HC PL APO CS2 Imm Corr (oil, water, glycerol) WD 0.68 mm, #11506343, Leica Microsystems; FOV: 581.82 µm x 581.82 µm) with oil immersion on a Leica SP8 system based on a DMI6000CSB microscope, which is equipped with diode (405 and 561 nm), argon (458, 488, and 514 nm) and a HeNe (633 nm) laser as well as two PMTs and two HyDs (high sensitivity Hybrid Detectors). The LAS X software was used for image acquisition with the Leica presets for DAPI (excitation 405 nm, emission 430–550 nm, HyD) and GFP (excitation 488 nm, emission 503–603 nm, PMT).

For the validation experiment (Fig. 3), a HEK293 cell line (stably transfected with pEGFP-N3 and showing 100% eGFP expression after clonal selection under 0.4 mg/mL geneticin) was mixed with non-transfected (non-fluorescent) HEK293 cells at different ratios and seeded into a µ-slide 8-well (#80826, ibidi; 300 µL/well at 4 × 10^5^ cells/mL). After overnight incubation, cell nuclei were stained with Hoechst 33342 and images were acquired as described above.

### Virus infection assay

African green monkey kidney epithelial cells (Vero) were seeded in 12 well plates (#83.3921.005, SARSTEDT AG & Co. KG, Germany) at a density of 2 × 10^5^ cells per well. At 90% confluency, the cells were first incubated with unfractionated heparin (#375095, Calbiochem, Germany) at different concentrations for 1 h. Then herpes simplex virus type 1 having the gene for green fluorescent protein (GFP) integrated into its genome (HSV-1_GFP) was added at a multiplicity of infection (MOI) of 0.1 for 48 h. Cell nuclei were labelled by Hoechst 33342 (1 µg/mL in medium, #H1399, Thermo Fisher Scientific) for 10 min at room temperature and the cells were fixed by 1% formaldehyde (#1039992055, Sigma-Aldrich) for 30 min. After being washed with PBS, the cells were imaged using widefield epifluorescence microscopy as mentioned above (2.1), in which infected cells showed green fluorescence due to GFP expression.

### Analysis macro

**Fig. 2:**
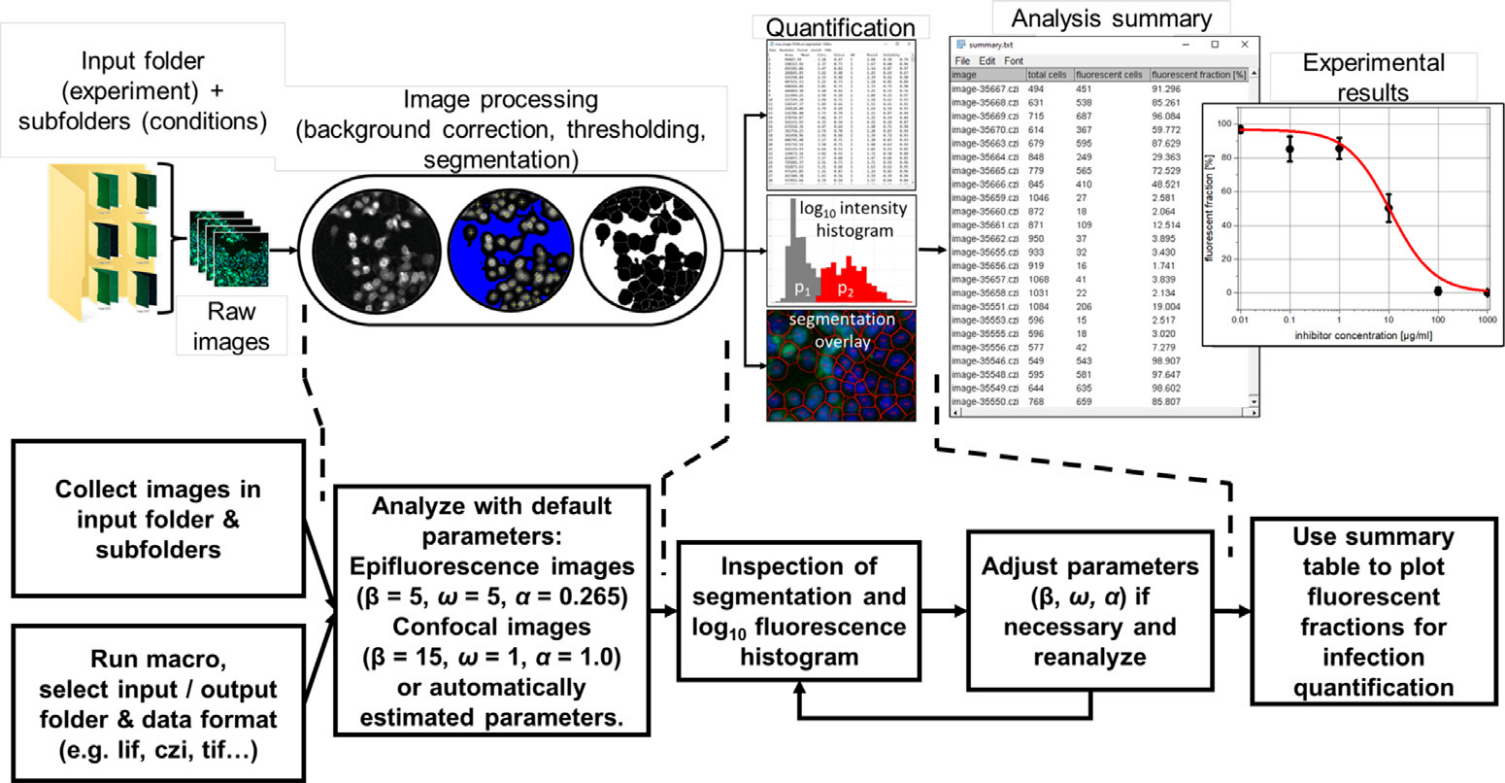
Usage flowchart of the analysis macro. All images in a user-selected input folder (and all subfolders) are automatically analyzed using default or automatically estimated analysis parameters (for details see Supplemental Materials Section S5: Automatic parameter estimation). The segmentation overlays and infection histograms are saved in a user-selected output folder. The analysis parameters can be adjusted after manual inspection of the analysis results in order to refine the analysis process until the segmentation overlay and intensity histograms show reasonable results.

Fig. 2 shows the basic steps for using the single-cell analysis macro presented in this work. All images to be analyzed must be collected in one input folder, which may contain several subfolders (*e.g.*, different inhibitor concentrations etc.). One output folder should be prepared, which will store all results generated by the macro. The macro is opened in Fiji [13] (which is an open-source distribution of ImageJ [14]) and started by pressing *Run*. The user then selects the input and output folder as well as the data format (without a “.”) of the input images (*e.g.*, lif, czi, tif) and defines, which channels contain the nucleus staining and the fluorescent marker signal, respectively (additional channels will be ignored). Afterwards, the user choses analysis parameters that are connected to the background threshold (denoted by β in the following), the segmentation sensitivity ω, and the intensity cutoff value α (for details see Supplemental Materials Section S4: Parameter optimization). These parameters can be optimized iteratively by repeating the analysis using revised parameters. For the initial analysis iteration, default parameters are implemented in the macro, which can be used in most cases. Alternatively, automatically estimated analysis parameters can also be used on demand (for details see Supplemental Materials Section S5: Automatic parameter estimation). The macro then automatically analyzes all selected images (taking approximately 1–2 s per image for a personal computer equipped with a CPU having a clock speed of 2.5 GHz, 2 cores, and 8 GB of RAM) and saves for each analyzed image the obtained segmentation overlay, the log_10_ histogram of the extracted cell intensity, a data table collecting various single-cell parameters (*e.g.*, area, mean intensity, x- and y-position, circularity, …) and a summary table to the output folder. The analysis workflow is also shown and explained further detail in a tutorial video (see Supplemental Materials Section S8).

To get a first impression on the validity of the parameters chosen for analysis, it is recommended to analyze either one image with two equally represented populations (non-fluorescent and fluorescent) or two images, in which most cells either exhibit fluorescence or not, which serve as positive and negative controls. A manual inspection of the segmentation overlay and the log_10_ intensity histogram of these images allows to check if analysis parameters need to be adjusted (see Supplemental Materials Section S4 for a guide for parameter optimization). The analysis can then be revised several times until the outcome of the segmentation and intensity analysis is acceptable for the user. Afterwards, the summary table (which contains the image titles, total cell number, and fluorescent cell number) can be used to quantify the property of interest, *e.g.*, transfection efficiencies or infection inhibition effects (for details see Supplemental Materials Section S1: Image processing steps).

### Method validation

**Fig. 3:**
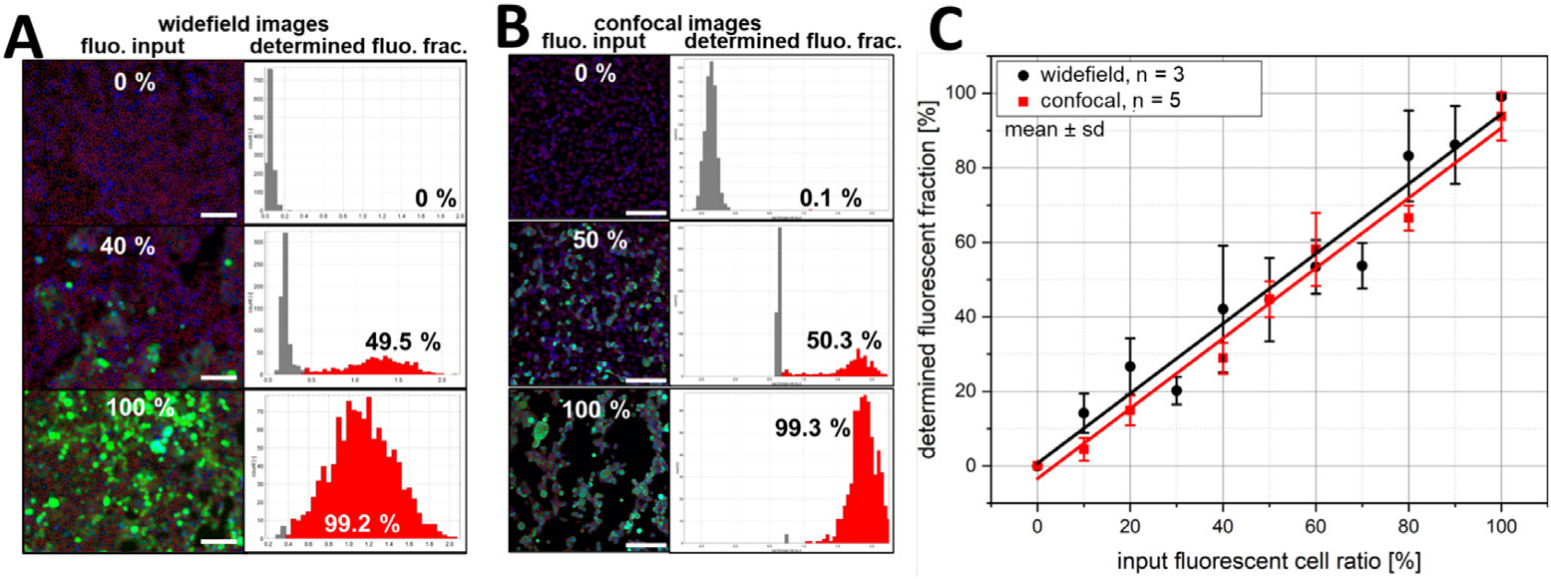
Validation by cell mixing. Two HEK293 cell populations, either expressing eGFP or not, were mixed at different ratios and three to five images per ratio were quantified using the analysis macro. Panels A and B show three representative microscopic images (scale bars = 150 µm), which were obtained using either widefield (A) or confocal microscopy (B), as well as the result of the segmentation process (red contours in the images) and the corresponding single-cell log_10_ intensity histograms. The fraction of fluorescent cells is indicated in the microscopy images (input values, defined by the mixing process) and histograms (extracted by single-cell analysis), respectively. For both imaging modalities a high correlation between input and determined fluorescent fraction is observed (panel C; widefield: *R*^2^ = 0.95, slope = 0.94 ± 0.06; confocal: *R*^2^ = 0.98, slope = 0.94 ± 0.05). The images of the widefield mixing series are provided in the Supplemental Materials Section (S9) so that the macro (with possible modifications) can be tested by the user. Sufficient analysis parameters are β = 15; ω = 5; α = 0.4.

The method implemented in the macro was validated by mixing an eGFP expressing HEK293 cell line with non-transfected HEK293 cells at different mixing ratios. Fig. 3A and B show representative images for three mixing ratios (0, 40 or 50, and 100% eGFP expressing cells; obtained using either widefield or confocal microscopy) as well as the results of the segmentation and the corresponding log_10_ intensity histograms obtained using the macro. For both imaging modalities, we observed a linear correlation between the determined and the input fluorescent fraction (Fig. 3C; widefield: *R*^2^ = 0.95, slope = 0.94 ± 0.06; confocal: *R*^2^ = 0.98, slope = 0.94 ± 0.05), indicating that the macro reliably determines the correct ratio of fluorescent and non-fluorescent cells. Only the widefield measurements having an input fraction of 30% and 70% of eGFP expressing cells showed a statistically significant deviation from the expected trend. However, these deviations were also present in the images and are therefore not due to a failure of the analysis macro. These deviations are attributed to minor manual errors in the initial cell mixing and random fluctuations in the number of cells that can be imaged in one field of view, as well as cells which have low expression levels [15].

### Use cases

#### Quantification of transfection efficiencies

**Fig. 4:**
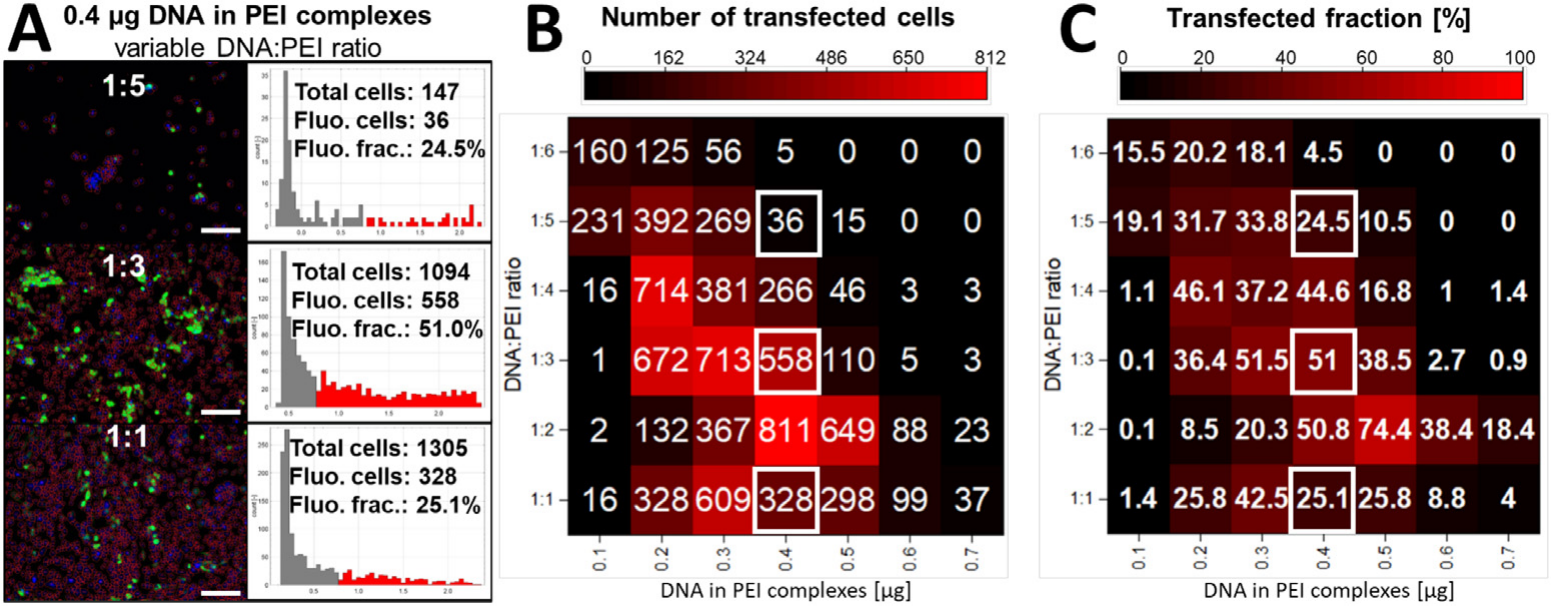
Quantification of transfection efficiencies of DNA:PEI complexes. HEK293 cells were transiently transfected with eGFP by PEI using different amounts of plasmid DNA (0.1–0.7 µg) and mixing ratios of DNA and PEI (1:1 – 1:6). Panel A shows three representative microscopic images of cells treated with 0.4 µg of DNA:PEI complexes (using DNA:PEI ratios of 1:5, 1:3, and 1:1 as indicated in the images) as well as the segmentation and corresponding single-cell log_10_ intensity histograms (scale bars = 150 µm). Panels B and C show heat maps that visualize the total number of transfected cells (B) or fraction of transfected cells in% (C), respectively. The three conditions shown in Panel A are marked with white squares in the heat maps. One image per condition was analyzed.

After successful validation, we applied the analysis macro to quantify the transfection efficiency of HEK293 cells, which were transiently transfected with eGFP for different transfection conditions (*i.e.*, for DNA:PEI ratios ranging from 1:1 to 1:6 and for a total amount of DNA:PEI complexes ranging from 0.1 to 0.7 µg). Fig. 4A shows three representative images corresponding to DNA:PEI ratios of 1:5, 1:3 and 1:1 (0.4 µg DNA:PEI complexes) as well as the obtained segmentation and corresponding log_10_ intensity histogram. For this series, the highest fluorescent fraction was observed for a DNA:PEI ratio of 1:3 (51%), so that an optimal transfection efficiency could be seen at this condition. The total number of observed cells decreased with decreasing DNA:PEI ratio, which is attributed to the well-known cytotoxic effect exhibited by PEI at higher concentrations (see Fig. S3 in the Supplemental Materials Section S3 - Plate reader validation) [16]. Hence, optimal transfection requires to find a balance between the DNA:PEI ratio (transfection efficiency) and the total PEI concentration (cytotoxicity).

To this end, we made use of the high-throughput capability of the macro and visualized the determined transfected cells (Fig. 4B) and transfected fraction for all tested conditions (Fig. 4C) as heat maps. A considerable amount of transfection (> 35%) was observed for DNA:PEI ratios ranging between 1:2 and 1:4 at a total amount of DNA:PEI complexes ranging between 0.2 and 0.5 µg. At lower ratios and amounts, less transfection was found, which indicates that the amounts of plasmid DNA or transfection reagent were too low. At higher ratios and amounts, the transfection efficiency was also reduced, which is again attributed to the cytotoxic effect of PEI at higher concentrations [16]. The highest fraction of transfected cells (75.4%) was found at a DNA:PEI ratio of 1:2 and a total amount of DNA:PEI complexes of 0.5 µg, which marks the optimal transfection condition in this setup if the transfected fraction is considered the key marker for transfection efficiency. Considering also cytotoxicity, the best balance between transfection efficiency and cytotoxic effects is observed at a DNA:PEI ratio of 1:2 and a total amount of DNA:PEI complexes of 0.4 µg.

#### Quantification of viral infection

**Fig. 5:**
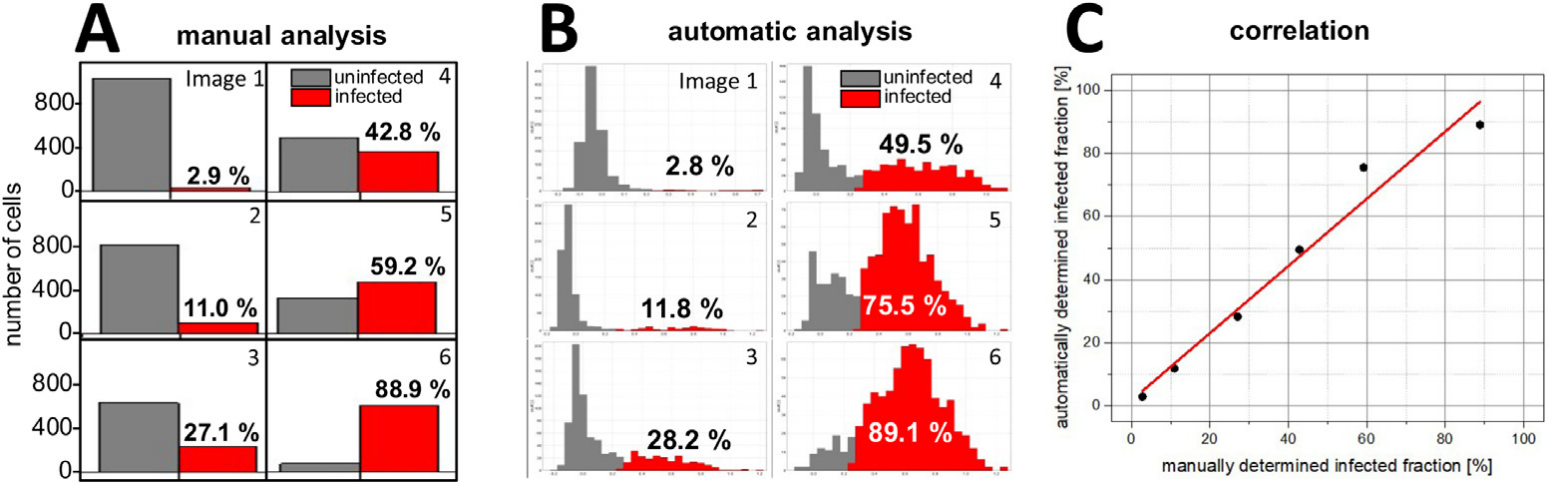
Comparison of manual and automatic quantification of the fraction of Vero cells that had been infected with a GFP-equipped herpes simplex virus 1 (HSV-1_GFP). Panel A and B show the fractions of uninfected (gray) and infected (red) cells as determined from six microscopic images. The values were derived by manual cell counting (A) or the single-cell log_10_ intensity histograms of the automatic analysis (B), respectively. The manually and automatically determined infected fractions show a very high correlation (*R*^2^ = 0.97, slope = 1.03).

In another use case, the analysis macro was used to automatically determine the fraction of Vero cells that had been infected with a variant of the herpes simplex virus 1 (HSV-1_GFP), which causes infected cells to exhibit green fluorescence due to GFP expression. Six images of infected cells that vary in their cell densities (approximately 650 to 1050 cells per image) and values of the infected fraction (approximately 2 to 90%) were chosen and analyzed manually (Fig. 5A) as well as with the analysis macro (Fig. 5B), which allowed to correlate automatically and manually obtained results (Fig. 5C). Cells were manually identified and counted based on their nucleus staining and their perinuclear space was inspected for green fluorescence to identify infected cells.

Similar to the validation using transfected HEK cells, we found a linear correlation (*R*^2^ = 0.97, slope = 1.03, Fig. 5C) between the manually and automatically determined fraction of infected cells. This indicates that both approaches give essentially identical results, with manual inspection being far more time-consuming than using the macro and providing no further information about fluorescence intensity distributions.

#### Quantification of virus infection inhibition

**Fig. 6:**
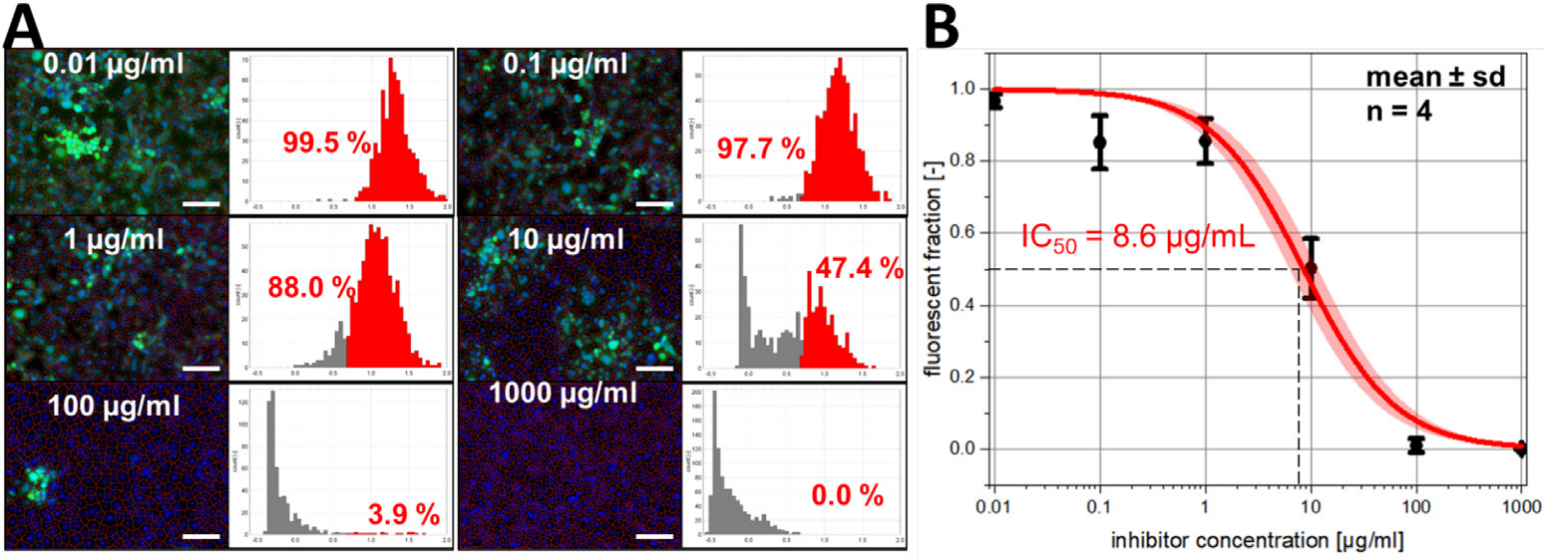
Quantification of the efficiency of heparin in inhibiting HSV-1_GFP infection of Vero cells. Panel A shows six representative images (corresponding to applied heparin concentrations of 0.01–1000 µg/mL as indicated; scale bars = 150 µm) together with the result of the segmentation and the corresponding single-cell log_10_ intensity histograms. The population of infected cells is shown in red in the histograms. The fraction of infected cells decreases with increasing heparin concentration (B), which is well described by a Langmuir-type inhibition model (solid line; red area indicates 95% confidence interval).

In addition to mere quantification of viral infection, our analysis macro can also be applied to quantify the efficiency of virus binding inhibitors [17]. This is demonstrated in Fig. 6, which summarizes the results of inhibition experiments, in which Vero cells were treated with heparin as inhibitor at different concentrations (0.01–1000 µg/mL) and infected with HSV-1_GFP. Fig. 6A shows six representative images and the corresponding log_10_ intensity histograms, in which the fluorescent (infected) cell fraction is indicated in red. The inhibitory effect of heparin can clearly be seen in the images, as well as in the log_10_ intensity histograms.

Fig. 6B shows the impact of the heparin concentration on mean value and standard deviation of the fluorescent (infected) cell fraction, which were calculated from four image replicates done for each concentration. This data allowed to determine the IC_50_ value (8.6 ± 1.3 µg/mL ∼ 573.3 ± 86.7 nM (M_W_ ∼ 15.000 ± 2.000 Da) of heparin inhibition, which was quantified by fitting the observed inhibition curve using the Langmuir-type inhibition model:

(1) $$f_{\inf}=\frac{1}{1+\frac{c_{\mathrm{inh}}}{IC_{50}}}$$

In this equation, *f_inf_* denotes the fraction of infected cells, which is observed at an inhibitor concentration *c_inh_*, while IC_50_ give the half maximal inhibitory concentration (*i.e.*, the inhibitor concentration, at which 50% of infection inhibition is observed). The determined IC_50_ value is in the range of previously reported IC_50_ values obtained by plaque reduction assays performed with Vero cells and similar HSV-1 variants (6–10 µg/mL [18], 240–380 nM [19]).

#### Discussion

In this work, we described the development, validation, and application of a fast and robust Fiji macro offering an automated fluorescence quantification with single-cell resolution. To ensure the versatility of the macro, it employs a watershed-based segmentation [20], which allows for thresholding of empty spaces without cells and avoids splitting of nuclei in cell-dense areas. This makes the macro applicable over a wide range of cell coverage (*i.e.*, for samples with low as well as high cell coverage). As not only the area of the nucleus is analyzed but the entire cell body (based on its autofluorescence signal), the macro can quantify fluorescence signals of the nucleus and the cytoplasmic area. We validated the macro for a wide range of fractions of fluorescent cells (between 0 and 99%), which makes it applicable to a broad spectrum of fluorescence images. We observed excellent agreement with manual image analysis when quantifying the fraction of transfected or virus-infected cells. Hence, the macro can be used to screen transfection efficiencies under different conditions as well as to quantify inhibition of virus infection of cells treated with virus inhibitors (providing the IC_50_ value of the inhibition process based on a quantification with single-cell resolution).

So far, the macro has been applied successfully on data derived using different cell lines (Vero, HEK293, HeLa), imaging techniques (widefield and confocal fluorescence microscopy) and labeling strategies (GFP transfection, virus protein surface staining), which demonstrates its feasibility. However, the user should be aware of the limitations of the method: The data quality is highly dependent on a sufficient segmentation based on staining of cell nuclei. If not all cell nuclei are sufficiently stained, the macro will not be able to correctly recognize them as cells and the resulting fraction of fluorescent cells will be incorrect. If cells are not in a monolayer and therefore overlap, the segmentation quality is reduced and nuclei in lower positions are excluded from the analysis, which causes a bias in the quantified populations. Also, shifts of the background intensity can cause a shift of the non-fluorescent cellular population in the log_10_ intensity histograms, which can lead to misclassification if the intensity cutoff is not carefully adjusted. Manual verification of the segmentation overlay and the log_10_ intensity histogram for each new experiment and condition is therefore recommended.

## Conclusion

Quantitative analysis of image data is a valuable complement to qualitative visual inspection for obtaining information about biological processes. The segmentation and analysis macro developed in this work provides a suitable tool for rapid quantification of fluorescence at the single-cell level, *e.g.*, for a quantification of cellular transfection, infection, or infection inhibition. It is based entirely on open-source components (contained in the Fiji package [13]) and allows to obtain accurate information with high throughput. The macro provided here (see Supplemental Material Section S7) performed well when validated by comparison with manually obtained image quantification data as well as in experiments, in which fluorescent and non-fluorescent cells were mixed in known ratios. The application of the macro presented is not limited to the use cases shown here; it is intended to be applicable for most monolayered cellular assays using nuclei staining and fluorescence as readout. As a Fiji macro, it is freely available and can be used and modified according to the users needs.

## CRediT authorship contribution statement

**Yannic Kerkhoff:** Methodology, Software, Validation, Formal analysis, Investigation, Data curation, Writing – original draft. **Stefanie Wedepohl:** Investigation. **Chuanxiong Nie:** Investigation. **Vahid Ahmadi:** Resources. **Rainer Haag:** Supervision, Writing – review & editing. **Stephan Block:** Supervision, Writing – review & editing.

## Declaration of Competing Interest

The authors declare that they have no known competing financial interests or personal relationships that could have appeared to influence the work reported in this paper.

The authors declare the following financial interests/personal relationships which may be considered as potential competing interests:

## Appendix Supplementary materials

Image, APPLICATION 1

Image, APPLICATION 2

Image, APPLICATION 3

Image, APPLICATION 3
