# Supplementary material for: A fast open-source Fiji-macro to quantify virus infection and transfection on single-cell level by fluorescence microscopy
Source: MethodsX. 2022 Sep 2;9:101834. doi: 10.1016/j.mex.2022.101834 (PMC9490200; doi:10.1016/j.mex.2022.101834)
Supplement: Supplementary file 1 [file mmc1.docx]

**Supplementary material**

## S1 Image processing steps

The main part of the macro consists of image processing steps (Fig. S1), which make the images accessible for a single-cell analysis based on Fiji’s *Particle Analyzer*. Fig. S1A shows a representative fluorescence image of HSV-1_GFP infected Vero cells (green), while cell nuclei are stained with Hoechst 33342 (blue). Non-infected cells barely show green fluorescence, but can be detected based on the nucleus staining. The blue and the green channels were color balanced (saturation of 0.35 % of pixels) to make the effect clearly visible to the naked eye. Infected cells can be identified by their bright green fluorescence. Also, a strong, inhomogeneous green background signal is present. To obtain quantitative information, the images are processed as follows:


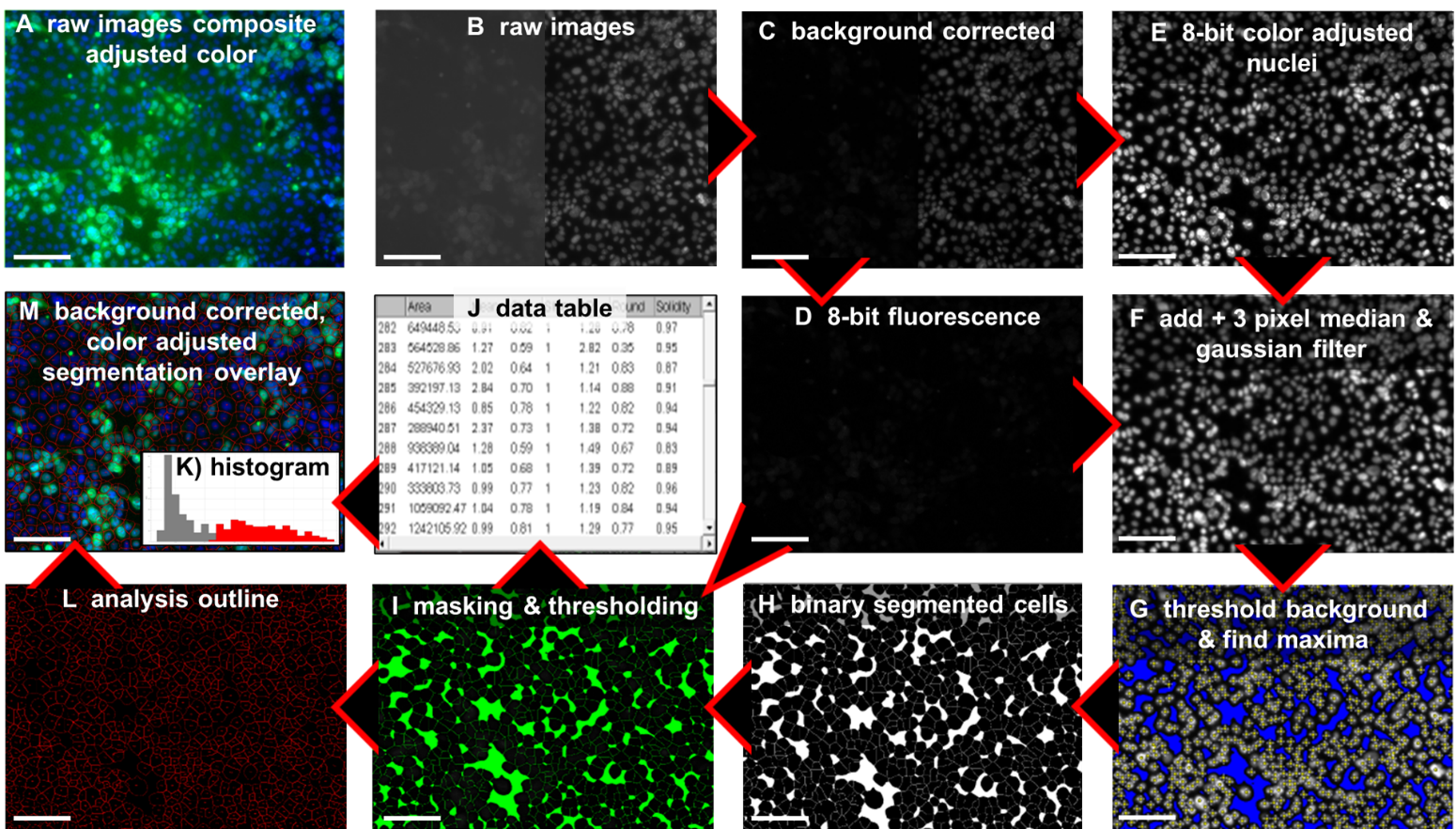


**Fig. S1.** Overview of the image processing steps (all scale bars = 150 µm). Panel A shows a representative color balanced composite fluorescence image of HSV-1_GFP infected (green) Vero cells with stained nuclei (blue). Panel B shows the raw images (left: GFP; right: nuclei) represented in gray values. Both channels are background corrected, using the rolling ball function with a 100-pixel radius (Panel C), converted to 8-bit format and split (Panel D and E). The nuclei channel is additionally color balanced. The fluorescence and nuclei channels are combined and a median and a Gaussian filter (both with a 3-pixel radius) are applied (Panel F). The background of the resulting image is thresholded (blue) and local intensity maxima (yellow) are detected (Panel G). Afterwards, the cells are segmented by a watershed-based segmentation and the resulting image is binarized (Panel H). The binarized image with the segmented cells is used as a mask on the 8-bit image of the green channel (Panel I) and the single-cell data is derived by the Particle Analyzer of Fiji (Panel J). Panel K shows the log_10_ intensity histogram indicating the population of dark (gray) and fluorescent cells (red) as determined from the single-cell data. The red outlines of analyzed cells, which are also delivered by the Particle Analyzer (Panel L), are overlaid with the background corrected and color balanced green and blue channels (Panel M).

First, the background of the green and blue channel is corrected by applying a rolling ball subtraction with a 100-pixel radius to remove illumination artifacts and inhomogeneities, which, especially in the green channel, would cause problems in the analysis. Afterwards, both image channels are converted to 8-bit resolution and split. The intensity of the nuclei channel is color balanced (saturation of 2 % of pixels) to make them more accessible for the identification of intensity maxima. The green channel is duplicated and added to the blue channel. A median and a Gaussian filter (3-pixel radius) are applied on the resulting image to remove noise pixels and minor inhomogeneities. The background is then thresholded with the beforehand chosen background threshold parameter β and nuclei are identified with the *Find Maxima* function with the prominence of the beforehand chosen segmentation sensitivity ω, which uses a watershed-based (Ref. 1) segmentation. The resulting binary image with the segmented particles is then inverted and used as a mask overlaid to the 8-bit fluorescence channel. The *Particle Analyzer* function is then called to generate the single-cell data from the segmented cells and the resulting data table is stored as a csv-file in the output folder. The mean intensity of the cells is then plotted as a log_10_ intensity histogram, in which the population above the intensity cutoff α is indicated in red. The name of the image file, the total cell number, the number of fluorescent cells, and the resulting fluorescent fraction (in %) is then added to the summary table, which is saved as a csv-file in the output folder. The outline of the analyzed particles is overlaid as red contours on the background corrected, color balanced green and blue channel. The histogram is stitched to the segmentation overlay and saved as a single png-file in the output folder.

## S2 Validation by cell mixing studies

**
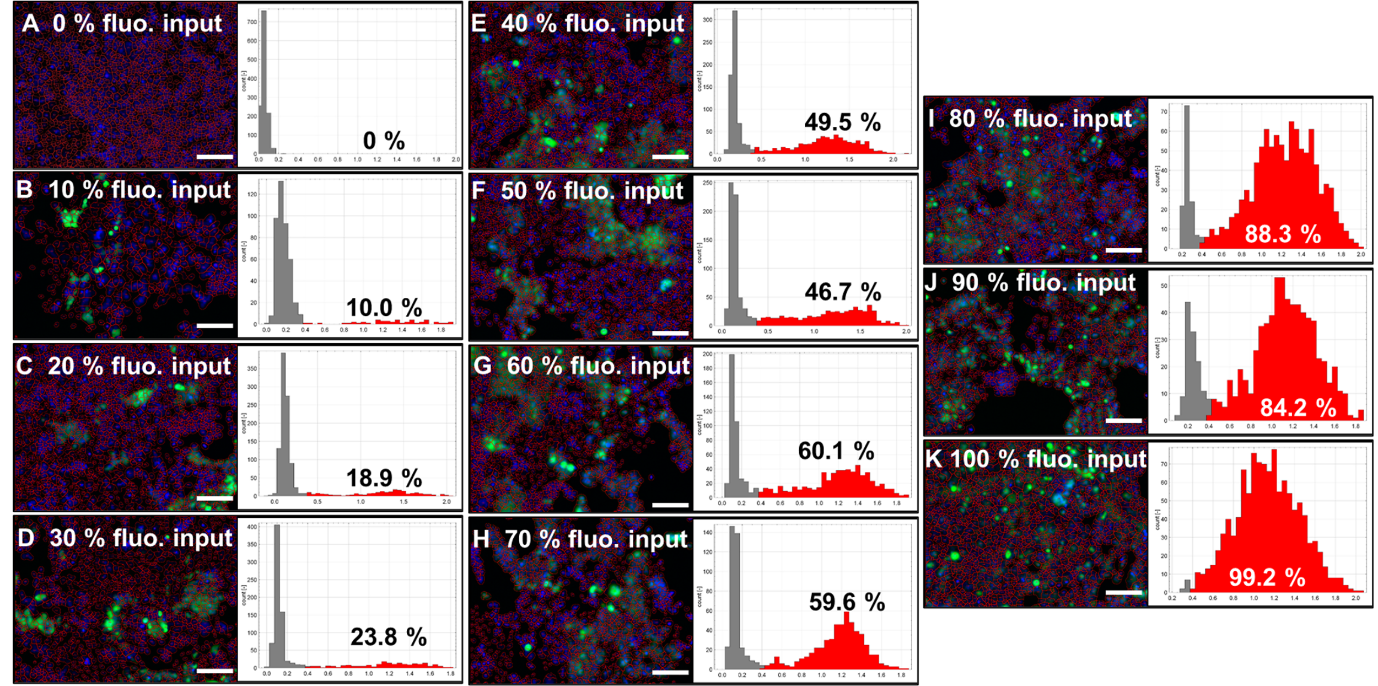
**

**Fig. S2.** Summary of all measurements of the mixing validation experiment (see Fig. 3 in the main manuscript). Shown are representative widefield images (scale bars = 150 µm) as well as the result of the segmentation process (red contours in the images) and the corresponding single-cell log_10_ intensity histograms. The fraction of fluorescent cells is indicated in the microscopy images (input values, defined by the mixing process) and histograms (extracted by single-cell analysis), respectively.

## S3 Validation using plate reader measurements

To validate the results obtained from the Fiji macro using an established system, the transfection series was also analyzed based on fluorescence data measured with a plate reader. Fig. S3 shows the results of the image analysis (Panels A to C) compared to the results obtained by a plate reader (Panels D to F). The number of detected cells (Fig. S3A) and fluorescence signal of the Hoechst staining (Fig. S3D) show a very similar pattern across the wells. In general, the number of cells decrease with increasing PEI concentration, which is attributed to toxicity. However, the cell number is slightly increased when 0.2 µg DNA was complexed with PEI at a ratio of 1:2 and 0.3 µg DNA was complexed with PEI at a ratio of 1:3, which is attributed to the cell attachment promoting feature of PEI (Ref. 2). In this experiment, an unusually high intensity of the Hoechst signal was found in the plate reader experiment at the condition of 0.2 µg complexes with a DNA:PEI ratio of 1:1, but not in the corresponding single-cell measurement. This could be due to a local cell aggregation or contamination that causes a misrepresentation of the total cell number by mere average fluorescence in a well plate and demonstrates the advantage of single-cell image analysis, in which aggregates or artifacts in the field of view can be identified easily. The number of fluorescent cells (Fig. S3B) determined by the macro as well as the GFP fluorescence signal (Fig. S3E) determined by the plate reader showed a very similar pattern as well. Both indicated optimal transfection efficiencies at DNA:PEI ratios ranging between 1:2 to 1:3 and at a total amount of DNA:PEI complexes of about 0.4 to 0.5 µg. The highest transfection efficiency is found at a DNA:PEI ratio of 1:2 and a total amount of DNA:PEI complexes of 0.4 µg if the total number of fluorescent cells (n = 811) is considered as a key marker. When the fluorescent fraction (Fig. S3C) is chosen as a key marker, optimal transfection efficiency is reached at a DNA:PEI ratio of 1:2 and a total amount of DNA:PEI complexes of 0.5 µg (74.4 %).

**
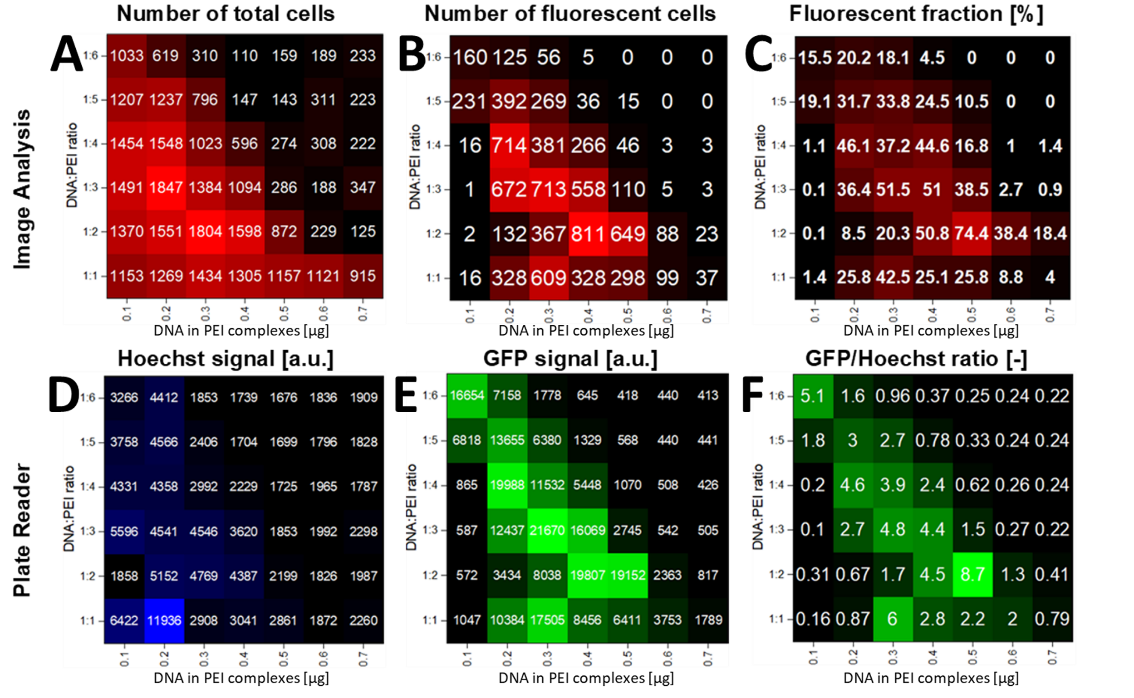
**

**Fig. S3.** Comparison of the results of the single-cell image analysis with plate reader measurements. Shown are heat maps of HEK293 transfection series derived by the single-cell image analysis (top row) and a plate reader measurement (bottom row), respectively. The heat maps visualize the total number of cells (Panels A and D), the total number of fluorescent cells (B, E) and the fraction of fluorescent cells (C, F), which were either derived directly by single-cell counting (A - C) or estimated by the intensity of the corresponding dye (D: Hoechst, E: GFP, F: GFP/Hoechst fluorescence ratio).

## S4 Guide to optimize the analysis parameters

Fig. S4 shows a guideline to optimize the analysis parameters β (background intensity threshold), ω (cellular segmentation sensitivity) and α (fluorescence intensity cutoff) by manual inspection of the segmentation overlay (for β and ω) and of the log_10_ intensity histogram (for α).

**
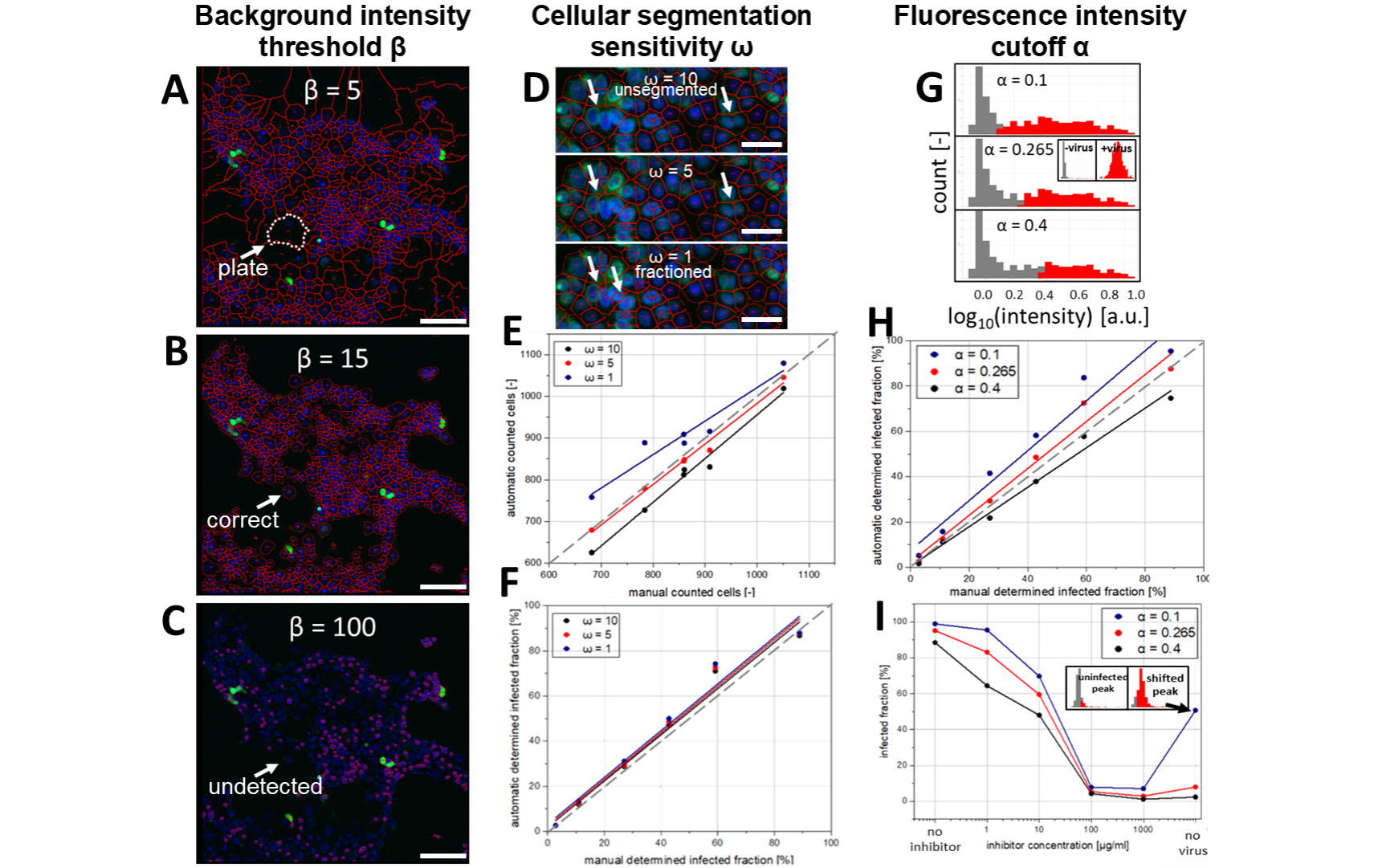
**

**Fig. S4.** Guidelines for the analysis parameter optimization. Panels A to C show the influence of three different values of the background threshold parameter (β) on the segmentation of eGFP-transfected HEK293 cells (scale bars = 100 µm). A β of 5 is too low and the background is not correctly thresholded. Cells in confluence are well segmented, while cells on the edges are mis-segmented, causing big parts of empty areas to be regarded as cell bodies. A β of 15 thresholds the background correctly and the cells are well segmented. A β of 100 is far too large, as only the brightest nuclei centers are identified and all others are thresholded with the background. Panels D to F show a comparison of manual and automatic cell counting and classification at different segmentation sensitivities (ω) using HSV-1 infected Vero cells. Panel D shows a crop of an infection assay image with cell segmentation using low (ω = 10), medium (ω = 5), and high (ω = 1) sensitivity (scale bars = 50 µm). Panel E shows the impact of the segmentation sensitivity on the automatically determined cell number. At a low sensitivity (black), the R^2^ is 0.98 and the difference from the manual counted cells is -6.2 ± 2.3 %. At a medium sensitivity (red), the R^2^ is 0.99 and the difference from the manual counted cells is -1.5 ± 1.4 %. At a high sensitivity (blue), the R^2^ is 0.93 and the difference from the manual counted cells is 6.2 ± 5.0 %. Panel F shows the influence of different segmentation sensitivities (ω) at constant intensity cutoff (α) on the determined infected fraction. All sensitivities show a high correlation of R^2^ ~ 0.97 and a minor deviation from the manual determined infected fraction (difference = -3.6 ± 5.4 %). Panels G to I show a comparison of the influence of different intensity cutoffs (α) used to determine the infected fraction in HSV-1_GFP infected Vero cells treated with an inhibitor (17). Panel G shows a log_10_ intensity histogram of an image, in which approximately half of the cells were infected, and highlights the impact of three different intensity cutoff values (α = 0.1, 0.265, and 0.4) on the assignment into infected (red) and non-infected cells (gray). The inset in the middle gives two additional histograms, which indicate the intensity distributions of non-infected and highly infected cells and enabled to determine the appropriate cutoff of 0.265. Panel H shows the impact of the intensity cutoff α on the determined fraction of infected cells. A cutoff value of 0.1 (blue) is too low and leads to overestimation of the infected fraction by 11.3 ± 8.3 %. A cutoff value of 0.265 (red) generates only a minor overestimation of 3.5 ± 5.3 % and is therefore appropriate. A cutoff value of 0.4 (black) is too high as the infected fraction is now underestimated by about -4.5 ± 5.3 %. All cutoffs show a high correlation of R^2^ > 0.94. Panel I shows the influence of the cutoff based determined infected fractions (mean of four images) of an inhibition experiment. All cutoff values show an inhibition effect with increasing inhibitor concentration. A cutoff value of 0.4 (black) underestimates the fraction of infected cells, while a value of 0.1 (red) causes the infected fraction to be overestimated and misclassifies some uninfected cells as infected as the intensity histogram is slightly shifted to higher intensities (small histograms), which distorts the mean infected fraction for the non-infected control.

The background threshold parameter β is the intensity below which empty spaces of the image should be excluded from the analysis, while areas above this intensity should contain nuclei as well as (auto)fluorescence signal from cells. It plays a minor role if the whole image is densely covered with cells, but becomes important when cells are on the edge of cell clusters. Fig. S4A-C shows how different background threshold parameters influence the cell segmentation of eGFP-expressing HEK293 cells. In the representative image shown in Fig. S4A, a β of 5 was too low, and thus the empty area of the image was not thresholded and cells on the edge of the cluster were not correctly segmented. Such giant edge cells had a highly reduced mean intensity, as the low background intensity is present in a major area of the mis-segmented cell and thus cells with a bright fluorescence signal were not recognized as such. A β value of 15 was appropriate, as the background is successfully thresholded from the cells and the cells at the center and the edge of the cluster were sufficiently segmented. Larger β values (*e.g.*, 100) were too high and many cells with dim nuclei were excluded from the analysis, which generated a bias in the quantification of the fraction of transfected cells. While the determination of a suitable threshold parameter β is very important for the analysis quality, the segmentation overlay makes it easy to identify inappropriate values and allows for a straightforward adjustment.

To determine the reliability of the automatic analysis with respect to a manual evaluation, six images with different cell densities (approximately 650 to 1050 cells per image) and degree of infection (approximately 2 to 90 %) were chosen and manually analyzed (Fig. S4D-F). For this, cells were counted by hand using the nucleus staining and infected cells were identified by visual inspection of the green infection signal in their cytoplasm. The manually derived data was regarded as a reference and plotted against the result of the macro using three different segmentation sensitivities ω to check for correlation (R^2^). For all chosen ω, the segmentation was in general successful, but at ω value of 10 some cells remained unsegmented in clusters, while at ω of 1 some cells became fragmented. However, these differences were hard to see by the naked eye even with the segmentation overlay. Fig. S4E shows the influence of ω more clearly. A ω value of 5 (red) showed the best correlation and agreement with the manually determined cell number with just a slight underestimation by about -1.5 %. A high ω value (black) and thus low sensitivity underestimated the number of cells ( -6.5 % for ω = 10). A low ω value (blue) and thus high sensitivity overestimated the number of cells due to fragmentation (6.2 % for ω = 1). However, Fig. S4F shows that the accurate segmentation of cells, at least with the three tested ω values, was not that important to gain reliable results. All tested segmentation sensitivities showed very similar correlations (R^2^ ~ 0.97) and only minor difference (underestimation of ~ -3.6 %) to the manually determined fraction of infected cells. As long as the majority of cells are correctly segmented, some fragmented or clustered cells seemed not to bias the resulting fraction of infected cells.

However, Fig. S4G-I shows that the determination of a suitable value of the intensity cutoff α is crucial to get reliable infection and inhibition data. Fig. S4G shows three log_10_ intensity histograms of an image, in which approximately half of the cells were infected (resulting in a bimodal infection histogram), and three different values of the intensity cutoff α (0.1, 0.265, 0.4) applied. The intensity cutoff value of α = 0.265 was determined by a positive and negative control experiment, where either no or a high virus titer was applied to the cells. This led to images, in which either none or nearly all cells were infected. The infection histograms of those images showed distinguishable histograms were with α < 0.265 nearly no cells were identified as infected (~ 3 %) and with α > 0.265 nearly all cells were identified as infected (~ 98 %). An α value of 0.265 is thus the correct parameter value of this measurement. The other cutoff values of 0.1 and 0.4 led to an overestimation and underestimation of fraction of infected cells, respectively. Fig. S4H shows the effect of the three different intensity cutoff values on the automatic determination of the infected fraction, which is plotted against the manually determined infected fraction. All cutoff values showed a high correlation (R^2^ > 0.94) but also the expected overestimation of infection (11.3 %) in case of α = 0.1 and the underestimation (-4.5 %) for α = 0.4. The intensity cutoff α should therefore be chosen very carefully and evaluated throughout the analysis process in order to avoid a bias of the automatically determined fraction of infected cells. However, Fig. S4I shows that the inhibitory effect can still be resolved (if present), even if slightly inappropriate intensity cutoff values are used. When the intensity cutoff α was too low (0.1, blue) some uninfected cells were misclassified as infected, as seen in the mean infected fraction of cells without virus infection. We attribute this to low, but in this case important, changes in autofluorescence and background, shifting the log_10_ intensity histogram slightly but above the intensity cutoff of 0.1. However, this artifact was obvious and if recognized either the intensity cutoff should be increased generally or the images which lead to this artifact should be analyzed separately with an increased intensity cutoff or be excluded from further analysis.

**S5 Automatic parameter estimation**

In addition to the manual selection and optimization of the analysis parameters, there is also the option to use automatically estimated analysis parameters. Fig. S5A shows the analysis parameters dialog box with the checked option “use automatic parameters”. If this option is chosen, the three analysis parameters are estimated by the equations (2) – (4). These estimations are based on standard values which were found matching for many widefield and confocal images and are additionally scaled with the intensity distribution of the current image to be analyzed. While this automatic option performed well in most of the cases presented here, the general performance of the automatically estimated parameters cannot be guaranteed and depends on the particulars of the experiment.

The automatic background intensity threshold β_auto_ is estimated by the equation:

|  | $\beta_{\mathrm{auto}}= 5+mean(Int_{nuc+fluo}<10)$ | (2) |
| --- | --- | --- |

so that there is at least a background intensity threshold of five, with the mean intensity of the pixels with an intensity of ten or less (of the image with the saturated nuclei and the added fluorescence, Fig. S1F) added to it. The range of this estimation for the cases tested was between ~ 5 and ~ 15.

The automatic segmentation sensitivity ω_auto_ is estimated by

|  | $\omega_{\mathrm{auto}}= 0.5+\frac{50}{mean\left( Int_{nuc+fluo} \right)}$. | (3) |
| --- | --- | --- |

This equation follows the idea that the presence of more, and presumably, dense nuclei in the image leads to a higher mean intensity and hence requires a more sensitive segmentation. The range of this estimation was between ~ 0.5 and ~ 5.5 for the cases tested.

The automatic intensity cutoff parameter α_auto_ is estimated by

|  | $\alpha_{\mathrm{auto}}=\log_{10} (1+mean(Int_{fluo}<5))$. | (4) |
| --- | --- | --- |

In this case, the intensity cutoff parameter scales directly with the background fluorescence from the fluorescence reporter channel (without nuclei signal), estimated by the mean intensity of all pixels with an intensity of 5 or lower. The range of this estimated parameter was between 0.2 and 1.1 for most images tested. Figure S5B and C show an example of the scaling of the automatic intensity cutoff parameter α_auto_. Two images from the same experiment performed under the same imaging conditions, showed different grades of background intensity (Fig. S5B: Log_10_ intensity of the non-fluorescent population at ~ 0.15. Fig. S5C: non-fluorescent population at ~ 0.3.) The automatically estimated intensity cutoff (α_auto_) scales accordingly and is shifted from ~ 0.4 in Fig. S5B to ~ 0.55 in Figure S5C.

As validation of the estimation quality, the widefield cell mixing image series (Supplemental Materials Section S9) was analyzed with automatically and manually chosen parameters and the analysis results showed a high correlation (R^2^ = 0.99 for validation images obtained using widefield microscopy, see Fig. S5D; R^2^ = 0.98 for validation images obtained using confocal microscopy). This shows, that the automatically estimated analysis parameters can be used for both widefield and confocal images.


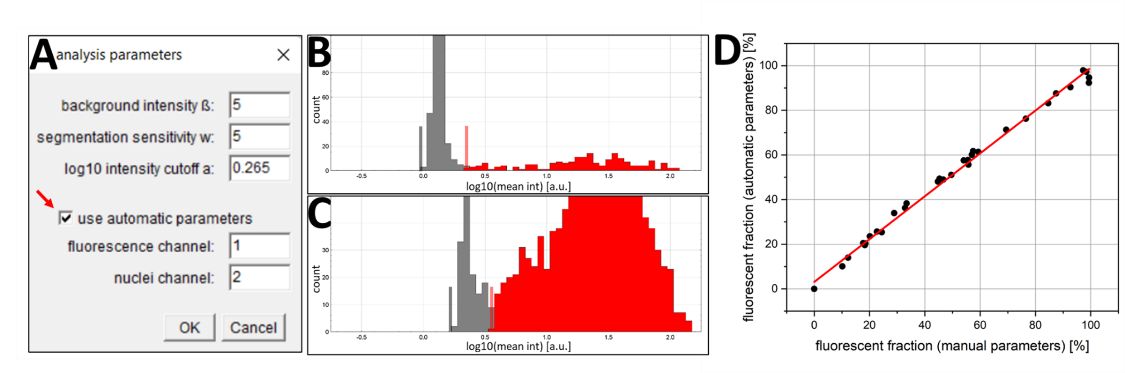


**Figure S5:** Overview of the option for automatically estimated analysis parameters. Panel A shows the analysis parameter dialog box with the selected checkbox “use automatic parameters”. Panel B and C show the log_10_ intensity histograms of two images. One having a non-fluorescent population around 0.15 (B), leading to an automatically estimated intensity cutoff (α_auto_) of ~ 0.4. The other histogram (C) has its non-fluorescent population shifted to ~ 0.3, which is accounted by an automatic shift of the intensity cutoff (αauto) to ~ 0.55. If this shift would not have been regarded in C, a significant fraction of the non-fluorescent population would have been misclassified as fluorescent. Panel D shows that the fractions of fluorescent cells, which were determined using manually and automatically chosen analysis parameters, show an excellent correlation (R^2^ = 0.99, slope = 0.96).

**S6 Image analysis software overview**

**Table S1:** Overview of the cited image analysis software (6-12). In columns 1 to 4, the short name of the software as well as the first author, the journal and the year of publication are given, respectively. Column 5 (Source(s)) lists the additional programs/software packages needed to run the analysis software. Column 6 (Scope and properties) gives a brief description of the software specialties. Columns 7 to 10 indicate whether the software is suitable for 2D, 3D, time-lapse and batch processing.

| **Method short name** | **Author** | **Journal** | **Year** | **Source(s)** | **Scope and properties** | 2D | 3D | time | batch |
| --- | --- | --- | --- | --- | --- | --- | --- | --- | --- |
| SCFQ | Kerkhoff *et al.* | MethodsX, this work | 2022 | Fiji | High-throughput single-cell fluorescence quantification (SCFQ) of 2D images of **cell monolayers** applicable for **varying cell density (up to confluence) and fluorescence distribution**. Watershed-based nuclei segmentation. Three analysis parameters must be chosen with the **option for an automatic parameter estimation.** | x |  |  | x |
| PiQSARS | Lévy *et al.* | MethodsX | 2020 | Fiji, RStudio, MATLAB | **Time-lapse focused** fluorescence microscopy analysis. Performs best at relatively low cell densities of 300 – 500 cells per cm^2^. | x |  | x | x |
| FNMM | Peterson *et al.* | Software Impacts | 2020 | Fiji | Quantification of **nuclei area with and without colocalization with fluorescence** signal. Two analysis parameters (2x threshold) are needed. Performs best at relatively low cell densities. | x |  |  |  |
| HASCIA | Chumakova *et al.* | Cytometry Part A | 2019 | ImageJ, Fiji, R, Shiny | **Quantitative assessment of protein expression** with single-cell resolution. Processing in ImageJ, data analysis in web application. Fluorescence assessment is based on nuclei mask. **Shading images needed** for every microscope/camera setup. Six analysis parameters must be chosen (2x threshold, 2x size, 2x circularity). | x |  |  | x |
| QuantIF | Handala *et al.* | Viruses | 2019 | ImageJ | **Counting nuclei** with and without **colocalization with fluorescence** signal. No quantification of fluorescence intensity. Three threshold parameters must be chosen. | x |  |  | x |
| Cytokit | Czech *et al.* | BMC Bioinformatics | 2019 | Python | Collection of open-source tools for quantifying and analyzing **properties of individual cells** in large fluorescent microscopy datasets with **spatial information**. | x |  |  | x |
| Infection Counter | Culley *et al.* | Viruses | 2016 | ImageJ | **Voronoi based nuclei segmentation** and fluorescence measurement to **quantify infection status**. Validated in infection fraction range up to 10 % infected cells. | x |  |  |  |
| FluoQ | Stein *et al.* | ACS Chemical Biology | 2013 | ImageJ, R | **Multiparameter fluorescence** image analysis applied to **oscillatory events** with **subsequent statistical analysis** of measured parameters in R. Only few input parameters needed. Cell **segmentation not based on nuclei** staining but on fluorescence reporter. | x |  | x | x |

**S7 Macro-script**

**S8 Video tutorial**

**S9 Widefield cell mixing image series**

**References**

1. L. Shafarenko, M. Petrou, J. Kittler, Automatic watershed segmentation of randomly textured color images, IEEE Transactions on Image Processing 6, 1530-1544 (1997).

2. A. Vancha, S. Govindaraju, K. Parsa, M. Jasti, M. González-García, R. Ballestero, Use of polyethyleneimine polymer in cell culture as attachment factor and lipofection enhancer, BMC Biotechnology 4, 23 (2004).
